# Nickel–Cobalt Hydroxides with Tunable Thin-Layer Nanosheets for High-Performance Supercapacitor Electrode

**DOI:** 10.1186/s11671-021-03543-w

**Published:** 2021-05-12

**Authors:** Luomeng Zhang, Hui Xia, Shaobo Liu, Yishan Zhou, Yuefeng Zhao, Wenke Xie

**Affiliations:** 1 School of Physics and Electronics, Central South University, Changsha, 410083 China; 2 Collaborative Innovation Center of Light Manipulations and Applications, Shangdong Normal University, Jinan, 250358 China

**Keywords:** Hydrothermal method, Nickel–cobalt double hydroxides, Nanosheets, High performance, Supercapacitor

## Abstract

Layered double hydroxides as typical supercapacitor electrode materials can exhibit superior energy storage performance if their structures are well regulated. In this work, a simple one-step hydrothermal method is used to prepare diverse nickel–cobalt layered double hydroxides (NiCo-LDHs), in which the different contents of urea are used to regulate the different nanostructures of NiCo-LDHs. The results show that the decrease in urea content can effectively improve the dispersibility, adjust the thickness and optimize the internal pore structures of NiCo-LDHs, thereby enhancing their capacitance performance. When the content of urea is reduced from 0.03 to 0.0075 g under a fixed precursor materials mass ratio of nickel (0.06 g) to cobalt (0.02 g) of 3:1, the prepared sample NiCo-LDH-1 exhibits the thickness of 1.62 nm, and the clear thin-layer nanosheet structures and a large number of surface pores are formed, which is beneficial to the transmission of ions into the electrode material. After being prepared as a supercapacitor electrode, the NiCo-LDH-1 displays an ultra-high specific capacitance of 3982.5 F g^−1^ under the current density of 1 A g^−1^ and high capacitance retention above 93.6% after 1000 cycles of charging and discharging at a high current density of 10 A g^−1^. The excellent electrochemical performance of NiCo-LDH-1 is proved by assembling two-electrode asymmetric supercapacitor with carbon spheres, displaying the specific capacitance of 95 F g^−1^ at 1 A g^−1^ with the capacitance retention of 78% over 1000 cycles. The current work offers a facile way to control the nanostructure of NiCo-LDHs, confirms the important affection of urea on enhancing capacitive performance for supercapacitor electrode and provides the high possibility for the development of high-performance supercapacitors.

## Introduction

As an environmentally friendly energy storage device, supercapacitor is attracting much attention owing to its unique advantages including long cycle life, superior reversibility, high power density and great reliability [1–3]. In recent years, it has been applied potentially in many prospective applications such as electric automobiles, smart wearable devices and portable computers, which is of great significance to future energy utilization and storage. However, the lower energy density of supercapacitors has always been an important reason for limiting its further development. It is well known that the crucial factor to determine capacity of supercapacitor is the electrochemical property of electrode material. Thus, the main solution at present is to develop electrode materials with excellent electrochemical properties [4–9]. The carbon materials [10–13], transition metal oxides [14], transition metal hydroxides (TMHs) [15–17] and conductive polymers [18] are the main materials used as supercapacitor electrodes. Among them, multihybrid nanomaterials of TMHs have become a strong trend of exploration because of the existence of synergistic effect, superior chemical composition controllability, outstanding redox activity and excellent anion exchange performance. As a typical TMHs, nickel–cobalt hydroxide is favored because of its low price, simple preparation process and high theoretical capacitance. However, it is still a big challenge to obtain high-performance electrode materials of nickel–cobalt hydroxides by a simple method.

The electrochemical properties of nickel–cobalt hydroxides depend largely on the special morphological nanostructures [19–23] and compositions of the metal ions [24, 25]. In previous studies, Wu et al. [26] synthesized vanadium-doped hierarchical porous nickel–cobalt layered double hydroxides nanosheet arrays that provided a high specific capacitance of 2960 F g^−1^ at a current density of 1 A g^−1^. Yan et al. [27] designed the nickel–cobalt layered double hydroxide hollow microspheres with hydrangea-like morphology, exhibiting a specific capacitance of 2158.7 F g^−1^ under a current density of 1 A g^−1^. Other efforts were made to decrease resistance, increase electrical conductivity of electrode materials and obtain other special morphologies with high specific surface area. When active material was grown on the surface of substrate, it would form a layered three-dimensional structure which could ensure the full contact between electrolyte ion and active material, and improve the reaction efficiency. Based on this, Ouyang et al. [28] obtained a high specific capacitance of 2047 F g^−1^ at a current density of 1 A g^−1^ by fabricating hierarchically structured spherical nickel–cobalt layered double hydroxides particles grown on biomass porous carbon. Zha et al. [29] successfully designed and fabricated highly open nickel–cobalt sulfide nanosheets on Ni foam, which presented low resistance and high specific capacitance of 2553.9 F g^−1^ under a current density of 0.5 A g^−1^. Unfortunately, although great progress has been made in the previous studies on nickel–cobalt layered hydroxides, the specific capacitance of the most of them as electrode materials still remains below 3000 F g^−1^.

In this work, we propose a facile and effective strategy to grow NiCo-LDHs on the nickel foam and adjust the nanostructures of electrode materials to improve charge storage capacity. The NiCo-LDHs are prepared by one-step hydrothermal process, with the structure including dispersity, thickness and porosities easily tuned through decreasing the content of urea under a fixed Ni to Co mass ratio of 3:1. The optimal NiCo-LDH-1 displays thin-layer nanosheets with the thickness of about 1.62 nm and obvious porous structures. The porous thin-layer structure can provide abundant active sites for the redox reaction, increase the affinity of the electrolyte and electrode materials, and reduce the diffusion resistance and migration distance of electrolyte ions. As a result, NiCo-LDH-1 exhibits an ultra-high specific capacitance of 3982.5 F g^−1^ under the current density of 1 A g^−1^, and high capacitance retention above 93.6% after 1000 cycles of charge and discharge at a high current density of 10 A g^−1^. The excellent electrochemical performance of NiCo-LDH-1 is further proved by assembling two-electrode asymmetric supercapacitor with carbon spheres, displaying the specific capacitance of 95 F g^−1^ at 1 A g^−1^ and the capacitance retention with 78% over 1000 cycles.

## Methods

The nickel foam (NF, 1 cm^2^) used in the experiment was provided by Canrd Co., Ltd., China. Prior to use, it was ultrasonicated in 2 M HCl for 15 min to remove the oxide attached on the surface and then washed by large amounts of deionized water and ethanol to remove ions from the surface. After that, it was dried at 60 °C for 3.5 h under vacuum. All other chemicals were of analytical grade, purchased from Sinopharm Chemical Reagent Co., Ltd., in China and used without further purification.

In a typical procedure, firstly 0.06 g NiCl_2_·6H_2_O and 0.02 g CoCl_2_·6H_2_O were dissolved in 80 ml deionized water under ultrasonication for 15 min. Secondly, urea was put into the mixed solution and kept ultrasonication for 10 min until the solid was completely dispersed. Then NFs with heat-resistant tape on one side were attached diagonally to the bottom of Teflon-lined stainless steel autoclave after measuring its mass. Finally, the homogeneous solution was transferred into the autoclave and then was kept at 100 °C for 8 h. After the reaction, the cooled NFs deposited with NiCo-LDHs were fetched out and washed with deionized water to remove impurities adhered on the surface and then dried at 60 °C for 4 h under vacuum condition. The total contents of urea were 0.0075, 0.015 and 0.03 g, respectively, corresponding to the samples of NiCo-LDH-1, NiCo-LDH-2 and NiCo-LDH-3. The sample, prepared in the same way as those mentioned above except without adding urea, was named as NiCo-LDH-0.

The X-ray automatic diffractometer (XRD, D8 Advance) was used to measure the crystal structure of materials. X-ray photoelectron spectrometer (XPS, ESCALAB 250Xi) was used to measure the element valence and content of materials. High and low vacuum scanning electron microscope (SEM, JSM-6360LV) was used to observe the morphology and composition of the microstructure of the surface of samples. Transmission electron microscope (TEM, TF20 Jeol 2100F) was used to observe the ultrastructure of the material. Atomic Force Microscope (AFM, Dimension Icon) was used to obtain the surface topography structure information and surface roughness information with nanometer resolution. Energy-dispersive X-ray spectroscopy element mapping (EDS mapping) was used to measure the distribution of elements.

A typical three-electrode system in 1 M KOH solution was used to test the electrochemical performance. The prepared nickel foam grown with electrode material was the working electrode, and the platinum plate and saturated calomel electrode were used as the counter electrode and the reference electrode, respectively, by which the cyclic voltammograms (CV), galvanostatic charge–discharge curves (GCD), electrochemical impedance (EIS) and cycle stability tests were measured. The specific capacitance *C*_c_ (F g^−1^) and specific capacity *Q* (C g^−1^) of the samples can be calculated according to the parameters obtained by galvanostatic discharge curves, and the expressions are as follows:

1 $$\begin{array}{c}{ C}_{c}=\frac{I\times \Delta t}{\Delta V\times m}\end{array}$$

2 $$\begin{array}{c}Q=\frac{I\times \Delta t}{m}\end{array}$$

where *I* (A) represents the discharge current; Δ*t* (s) means the discharge time; Δ*V* (V) gives the discharge potential window; and *m* (g) corresponds to the mass of the active material, about 0.0012 g.

An asymmetric supercapacitor (ASC) is fabricated in a two-electrode system after balancing the charges by Q_s+_ m_s+_  = Q_s-_ m_s-_. The NiCo-LDH-1/NF is used as the positive electrode, and the negative electrode is obtained by mixing carbon spheres, carbon black and PTFE at the ratio of 8:1:1 on the NF. The electrolyte is the same as that in the three-electrode system, and the range of potential windows for ASC is 0 ~ 1.4 V. To examine the practical electrochemical performances, the specific energy density *E*_*c*_ (W h kg^−1^) and specific power density *P*_*c*_ (W kg^−1^) for the asymmetrical configuration are calculated as follows:

3 $$\begin{array}{c}{E}_{c}=\frac{{C}_{c}{\left(\Delta V\right)}^{2}}{2\times 3.6} \end{array}$$

4 $$\begin{array}{c}{P}_{c}=\frac{{E}_{c}\times 3600}{\Delta t}\end{array}$$

where *I* (A) represents the discharge current; Δ*t* (s) gives the discharge time; Δ*V* (V) corresponds to the potential windows; *m* (g) means the total active mass of the positive and negative electrodes, about 0.0065 g.

## Results and Discussion

**Fig. 1:**
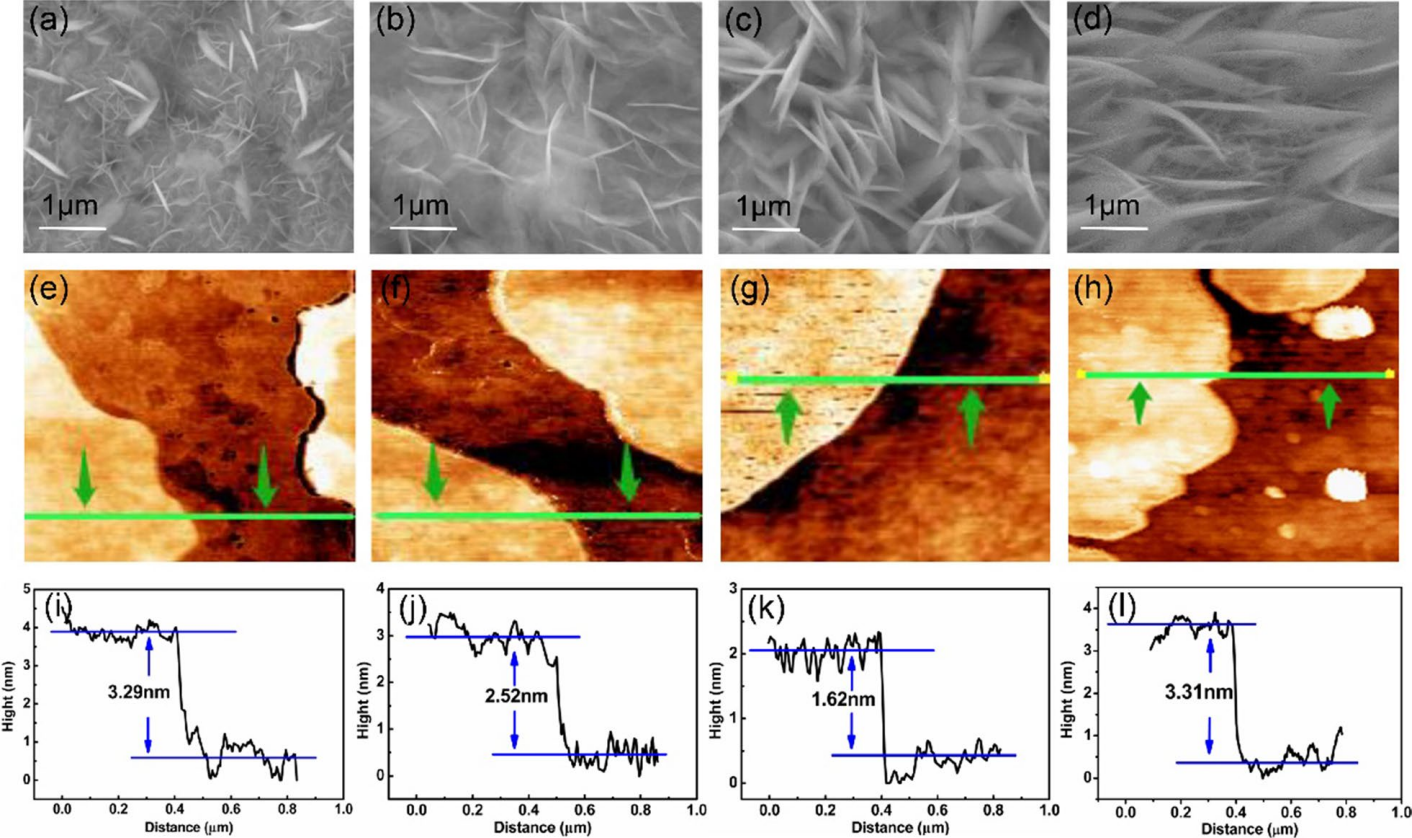
**a**–**d** SEM images of the samples: **a** NiCo-LDH-3, **b** NiCo-LDH-2, **c** NiCo-LDH-1, **d** NiCo-LDH-0; **e**–**h** AFM images of the sample: **e** NiCo-LDH-3, **f** NiCo-LDH-2, **g** NiCo-LDH-1, **h** NiCo-LDH-0; **i**–**l** the thicknesses of the samples: **i** NiCo-LDH-3, **j** NiCo-LDH-2, **k** NiCo-LDH-1, **l** NiCo-LDH-0

Figure 1 illustrates the microstructures and morphologies of NiCo-LDHs grown on the NF prepared with different content of urea. Figure 1a–l shows the SEM images, the AFM images and the thickness of the samples, respectively. As shown in Fig. 1a, the synthesized NiCo-LDH-3 displays sheet-like structures stacked and interwoven in the horizontal direction parallel to the NF. The sheet-like structures are uneven and have strong adhesion. With the urea content gradually decreases, the NiCo-LDHs gradually grow in the vertical direction and are perpendicular to the NF. As shown in Fig. 1c, when the content of urea is reduced to 0.0075 g, the nanosheets of NiCo-LDH-1 are interwoven and distributed on the surface of the NF, which forms an obvious three-dimensional structure and rich pore structures between the layers. The morphologies of these nanosheets are beneficial to increasing the specific surface area of the electrode to provide abundant reactive sites for the reaction [30]. Therefore, it can significantly increase the contact surface with the electrolyte to promote the electrochemical reaction for contributing large specific capacitance in the electrochemical reaction [31]. Figure 1e–l is the AFM images to detect the thicknesses of NiCo-LDHs nanosheets. For the samples of NiCo-LDH-3, NiCo-LDH-2 and NiCo-LDH-1, the corresponding thicknesses are 3.29, 2.52 and 1.62 nm, respectively. It is shown that the thickness of the nanosheets of the material decreases gradually with the content of urea decrease. The ultra-thin structure in NiCo-LDH-1 provides good conditions for the formation of the pore structure and shortens the distance to ion transfer. However, the SEM image of NiCo-LDH-0 (Fig. 1d) shows that the sample prepared without adding urea also displays sheet-like structures, but the thickness is 3.31 nm (Fig. 1h, l), which is thicker than those of other samples prepared with urea. It implies that the microstructures and morphologies of NiCo-LDHs can be affected by the content of the urea. In the process to obtain the samples involving in urea, the urea slowly decomposes into NH_3_ and CO_2_ at the high temperature and further produces CO_3_^2−^, NH_4_^+^ and OH^−^ ions by the reaction with water. Under the condition of lower content of urea, Co^2+^ and Ni^2+^ ions have few contact sites with OH^−^, which will form the thinner layer nanosheets structures [32]. Nevertheless, Etching does not occur during the process of the sample prepared without urea. As a result, compared with the samples obtained with urea, the thickness of the sample without adding urea becomes thicker.

**Fig. 2:**
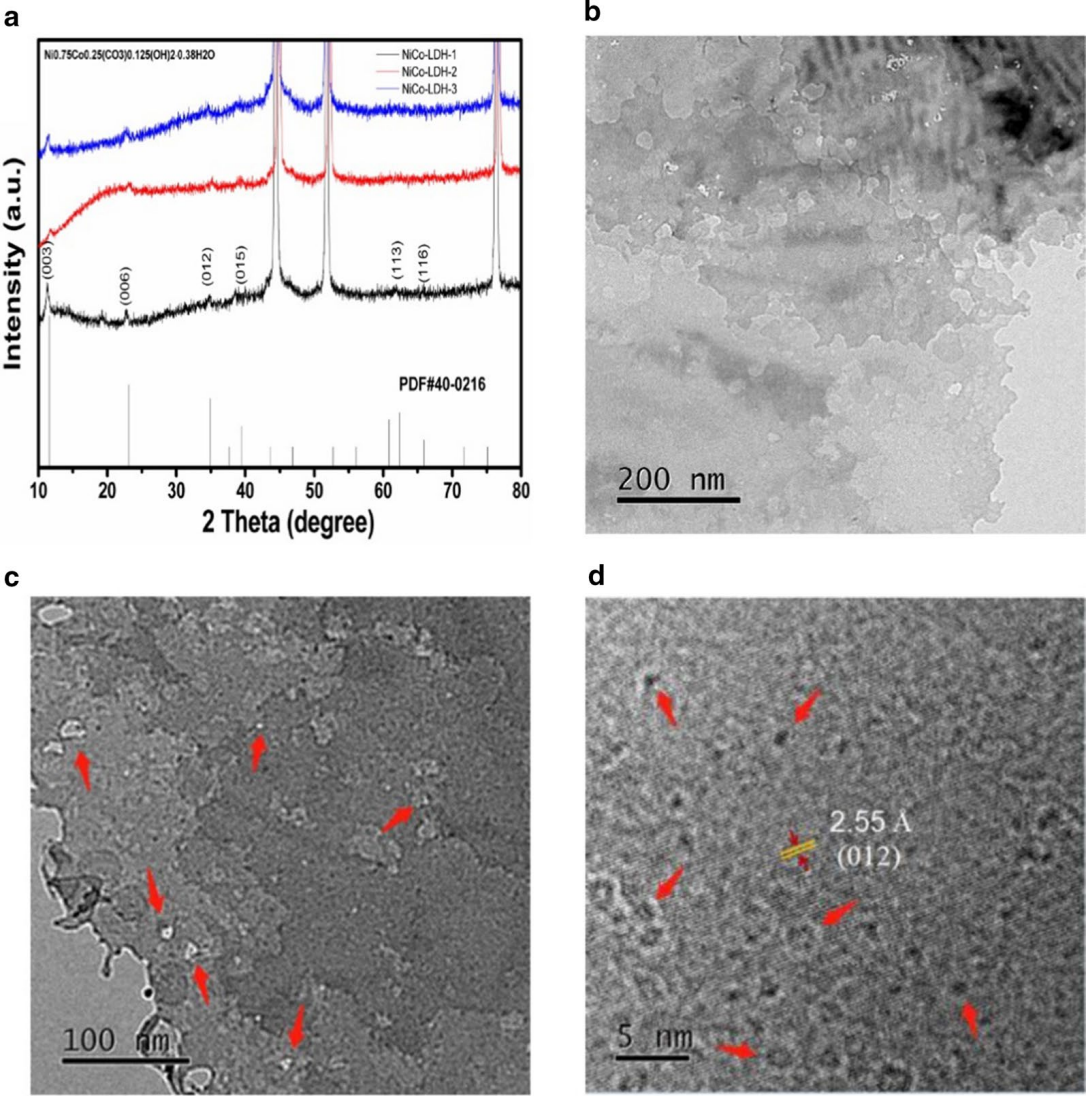
**a** X-ray diffraction patterns of the samples; **b**–**d** TEM images of NiCo-LDH-1

The XRD spectra of NiCo-LDHs are shown in Fig. 2a. After compared with the standard PDF card, all materials can be assigned to Ni_0.75_Co_0.25_(CO_3_)_0.125_(OH)_2 0.215_·0.38H_2_O (PDF#40–0216). The diffraction peaks at 2θ of 11.59°, 23.14°, 34.95°, 39.40°, 62.44° and 65.96° correspond to nickel–cobalt hydroxides (003), (006), (012), (015), (113) and (116) crystal planes, respectively. The detail micro-morphology of NiCo-LDH-1 is further characterized by TEM. As is shown in Fig. 2b–d, NiCo-LDH-1 appears as the thin porous layers and there is very little stack between layers. That is because the reduced urea content improves the dispersibility of the material and reduces the lateral stacking between the layers. The three-dimensional growth structure makes the sheet structure of the material thinner and has obvious pores. The existence of the thin-layer porous structure can greatly increase the immersing of electrolyte into the electrode material, reduce the diffusion resistance and migration distance of electrolyte ions [33].

**Fig. 3:**
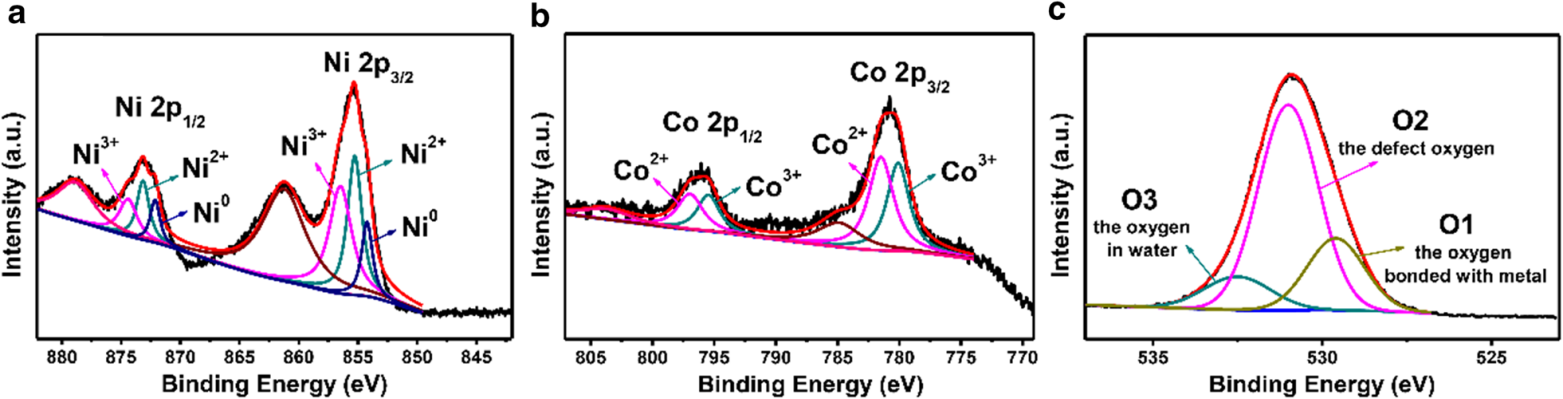
X-ray photoelectron spectra of **a** Ni 2p, **b** Co 2p and **c** O 1 s of NiCo-LDH-1

Figure 3 shows the XPS analysis of the NiCo-LDH-1. For the Ni 2p XPS spectrum in Fig. 3a, two main peaks are observed around 873.1 and 855.5 eV corresponding to Ni 2p_1/2_ and Ni 2p_3/2_, respectively. The peaks with binding energy at 874.4 and 856.5 eV are attributed to Ni^3+^, while the fitting peaks at 873.1 and 855.3 eV correspond to Ni^2+^ [34, 35]. Meanwhile, the peaks at 872.1 and 854.2 eV imply the presence of Ni^0^ which are ascribed to NF and other two peaks at 878.8 and 861.2 eV can be assigned to satellite peaks. Similarly, Fig. 3b depicts the fitted XPS spectrum of Co 2p, and two main peaks located at 796.1 and 780.8 eV are indexed to Co 2p_1/2_ and Co 2p_3/2_, respectively. The doublets at 796.9 and 781.5 eV agree with Co^2+^, while the other doublets at 795.5 and 780.1 eV are consistent with Co^3+^ [35, 36]. The corresponding satellite peaks are at 784.9 and 803.7 eV. The O 1 s spectrum is shown in Fig. 3c, in which the peaks centered at 529.6, 531 and 532.5 eV should be assigned to the oxygen bonded with metal (O1), the defect oxygen (O2) with low coordination and the oxygen in water (O3) that is physically and chemically bonded on and within the surface, respectively [35]. These results show that the NiCo-LDH-1 has rich distribution of valence states, which is beneficial to the improvement of electrochemical performance.

**Fig. 4:**
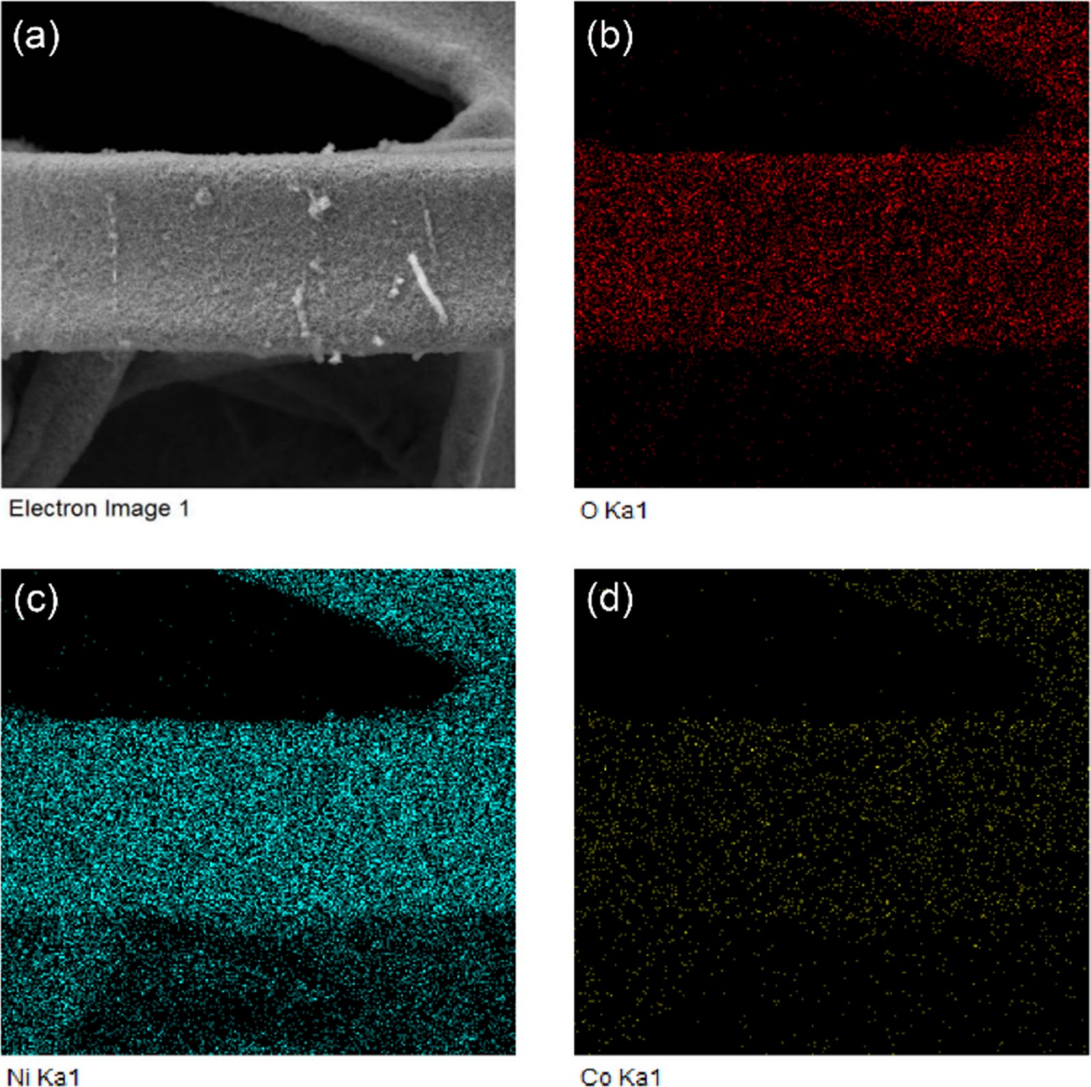
**a** SEM of NiCo-LDH-1; EDS element mapping diagrams of **b** Ni, **c** Co and **d** O in NiCo-LDH-1

The EDS mapping diagrams of NiCo-LDH-1 are shown in Fig. 4a–d. It can be seen from the figures that the Ni, Co and O elements are uniformly distributed over the material, which is in accordance with the results of XPS.

**Fig. 5:**
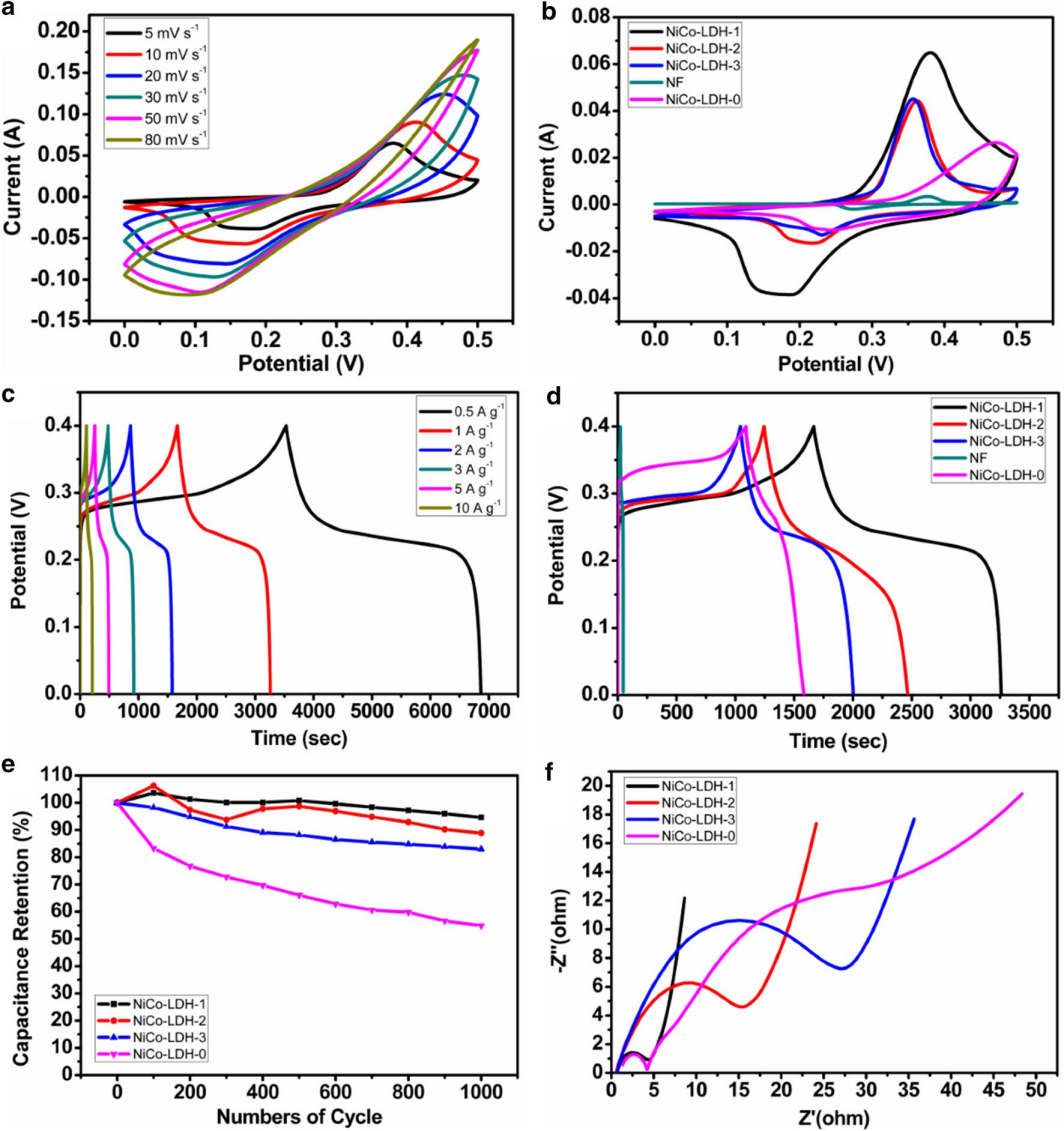
**a** CV curves of NiCo-LDH-1 at different scan rates; **b** CV curves of samples at scan rate of 5 mV s^−1^; **c** GCD curves of NiCo-LDH-1 at different current densities; **d** GCD curves of samples at 1 A g^−1^; **e** cyclic stability diagram of NiCo-LDH-1, NiCo-LDH-2, NiCo-LDH-3 and NiCo-LDH-0 at 10 A g^−1^; **f** Nyquist plots of NiCo-LDH-1, NiCo-LDH-2, NiCo-LDH-3 and NiCo-LDH-0

In order to test the electrochemical performances of NiCo-LDHs, the CV, GCD EIS and cycle stability tests are carried out in a typical three-electrode test system. Figure 5a shows the cyclic voltammetry curves of NiCo-LDH-1 at different scan rates. It can be seen that there are obvious oxidation peaks and reduction peaks observed for all the samples, and the areas for anodic and cathodic peaks at a fixed scan rate are the same basically, which indicates that the electrode material has excellent reversibility. The redox reactions can be expressed as:

5 $$\begin{array}{c}{Ni\left(OH\right)}_{2}+{OH}^{-}\leftrightarrow NiOOH+{H}_{2}O+{e}^{-}\end{array}$$

6 $$\begin{array}{c}{Co\left(OH\right)}_{2}+{OH}^{-}\leftrightarrow CoOOH+{H}_{2}O+{e}^{-}\end{array}$$

7 $$\begin{array}{c}CoOOH+{OH}^{-}\leftrightarrow Co{O}_{2}+{H}_{2}O+{e}^{-}\end{array}$$

Figure 5b presents the cyclic voltammetry curves of NiCo-LDHs at the scan rate of 5 mV s^−1^. It can be seen that the oxidation and reduction peak currents of the NiCo-LDH-1 are significantly higher than those of the NiCo-LDH-2, the NiCo-LDH-3 and the NiCo-LDH-0, and the area enclosed by the peak width and peak current intensities of NiCo-LDHs increases sequentially. According to the previous literature [37], the area enclosed by the curve can reflect the contribution of the material to the capacitance. The larger the integral area enclosed under the same scan rate and voltage window, the better the capacitance performance of the material, so the regulated NiCo-LDH-1 by decreasing urea content makes the capacitance performance better. In addition, it can be observed from the CV curves of NF at 5 mV s^−1^ that the area of CV curves for NF is negligible compared to other samples, which indicates that the capacitance contribution of NF is insignificant.

The galvanostatic charge and discharge curves of NiCo-LDH-1 at different current densities are shown in Fig. 5c. Obviously, NiCo-LDH-1 has an ultra-high specific capacitance of 4166 (1667 C g^−1^) and 3982.5 F g^−1^ (1593 C g^−1^) at a current density of 0.5 and 1 A g^−1^, respectively. At the strong current density of 10 A g^−1^, the specific capacitance of 2550 F g^−1^ (1020 C g^−1^) can be still retained. By comparing with the results of literature as shown in Table 1, our work is more advantageous.

**Table 1:** Comparison of the performance of our work with those in literature

Active materials	Electrolyte	Current density (A g^−1^)	Specific capacitance (F g^−1^)	Capacitance retention (%)	Refs.
NiCoV-LDH NSAs	6 M KOH	1	2960	81.8	[26]
Ni/Co-LDH	6 M KOH	1	2158.7	97.5	[27]
NiCo-LDHs	6 M KOH	1	2047	75.8	[28]
NiCo-S NiCo-LDH/rGo CS/NiCo-CH (NiCo-LDH)S_HH_ Ni-Co DHs/EG PANI/NiCo-LDH NAC/NiCo-DHs NiCo-LDH	2 M KOH 2 M KOH 1 M KOH 6 M KOH 6 M KOH 2 M KOH 6 M KOH 1 M KOH	0.5 1 1 1 1 0.5 1 1	2553.9 1675 643 1765 1246 1845 1920 3982.5	92 81.2 84.2 86 80.1 82 95.54 93.6	[29] [38] [39] [40] [41] [42] [43] This work

Figure 5d is the galvanostatic charge and discharge curves of NiCo-LDHs under the same current density and voltage window. It can be observed that when the urea content decreases, the charge and discharge times of NiCo-LDHs become longer in turn. The specific capacitance changes from 2405 F g^−1^ (962 C g^−1^) for the NiCo-LDH-3 to 3052.5 F g^−1^ (1221 C g^−1^) for the NiCo-LDH-2 and finally increases to 3982.5 F g^−1^ (1593 C g^−1^) for the NiCo-LDH-1. It illustrates that the change of urea content has important influence on the redox reaction of materials. The reason is that with a high urea content, the NiCo-LDHs mainly grows in the direction parallel to NF surface laterally, and the layers are stacked together, which increases the holistic thickness of the layers, so that the electrolyte cannot penetrate well and redox reactions resulting in the pseudocapacitance can only carry out on or near the surface. The decrease of urea content makes the dispersibility of the material better. The NiCo-LDHs gradually gets rid of the stacked state between the layers. The three-dimensional growth structure makes the layers structures of the material thinner and the pores obvious. This provides more active sites for the reaction and reduces the diffusion resistance and migration distance of electrolyte ions, which is conducive to the transmission and diffusion of ions, thereby greatly improving the pseudocapacitance performance of the material [36, 44]. According to the GCD curve of NiCo-LDH-0, the specific capacitance of the sample is 1232.5 F g^−1^ (493 C g^−1^) at a current density of 1 A g^−1^ which is lower than those of the samples obtained with urea. It further confirms that the changed structure including morphology and thickness caused by the introduction of urea has a positive promotion effect on the electrochemical properties of NiCo-LDHs.

Figure 5e shows the cycle stability of the NiCo-LDHs. Under the current density of 10 A g^−1^, the capacitance retention rate of NiCo-LDH-1 is above 93.6% after 1000 cycles, higher than 88.9% and 83% for the NiCo-LDH-2 and the NiCo-LDH-3, respectively. However, the capacitance retention rate of the NiCo-LDH-0 is only 54.9%. It indicates that the suitable urea content can effectively improve the stability of electrode materials. Moreover, during the 100 to 500 cycles, the capacitance retention of the NiCo-LDH-1 is greater than 100%, which suggests the thinner vertical layer structure during this cycle process can sufficiently make the electrolyte diffuse to the near-surface of active substance to support the process of redox reaction. Figure 5f is the result of EIS test for the NiCo-LDHs. The Nyquist plots are composed of two parts: high-frequency and low-frequency region, corresponding to a semicircle and a line section, respectively. The diameter of semicircle in high-frequency region reflects the electron transfer resistance. The smaller the semicircle diameter is, the smaller the electron transfer resistance will be. The slope of the line represents the diffusion ability of the electrolyte ions on the material surface. The higher the slope is, the stronger the diffusion ability will be [45]. For the samples of NiCo-LDH-1, NiCo-LDH-2 and NiCo-LDH-3, when the urea content gradually decreases, the transfer resistance and migration distance of electrons decrease for the corresponding electrodes, the transmission rate of ions to the electrode surface increases, and the conductivity of the material gradually improves. However, for the sample of NiCo-LDH-0, though the electron transfer resistance of that is relatively small, the transmission rate of ions is too slow to match electronic transmission capability, which leads to the poor electrochemical performance.

**Fig. 6:**
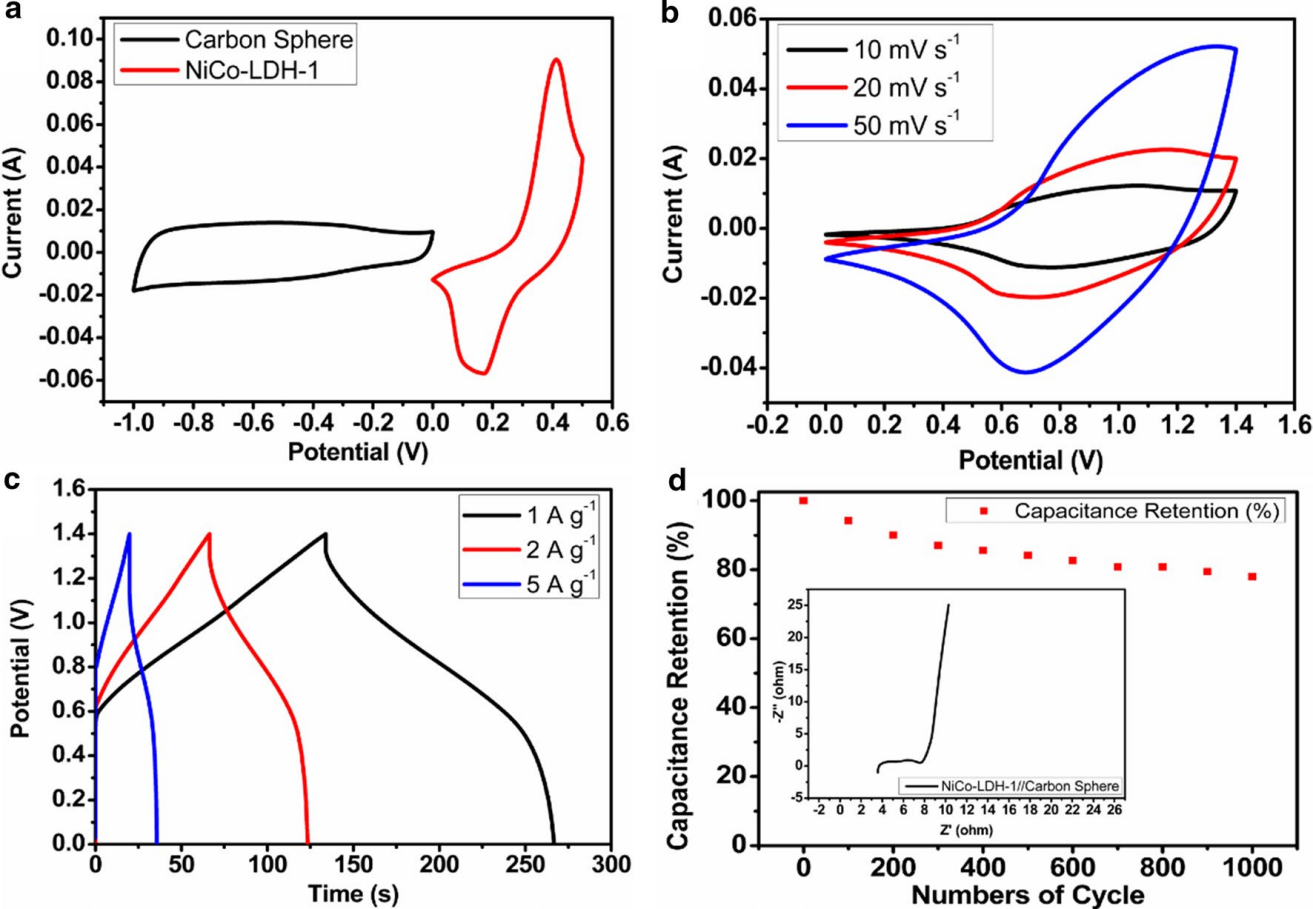
Electrochemical performance of the NiCo-LDH-1/carbon sphere asymmetric supercapacitor: **a** CV curves at scan rate of 10 mV s^−1^; **b** CV curves under different scan rates; **c** GCD curves at different current densities; and **d** cyclic stability under the current density of 10 A g^−1^

The excellent electrochemical performance of NiCo-LDH-1 as the positive electrode is further confirmed by fabricating two-electrode asymmetric supercapacitor with the carbon spheres as the negative electrode. Figure 6a is the CV curves of the carbon sphere and NiCo-LDH-1 electrodes at 10 mV s^−1^. The carbon sphere and NiCo-LDH-1 electrodes with the potential window of -1 ~ 0 and 0 ~ 0.5 V can be assembled into a stabilizing device with the extended voltage of 1.5 V effectively, as shown by the CV curves at 10 mV s^−1^ of the device in Fig. 6b.

After comparing Fig. 6a, b, there are two primary distinctions observed markedly between them in potential window and the CV curve shape. The causes for these differences lie in the following aspects: 1) Relative to the saturated calomel reference electrode, the voltage window of the single NiCo-LDH-1 and carbon spheres electrode is 0 ~ 0.4 and -1 ~ 0 V in a three-electrode system, respectively. However, after we use NiCo-LDH-1 as the positive electrode to fabricate two-electrode asymmetric supercapacitor with the carbon spheres as the negative electrode, the voltage window of the device is relative to the negative electrode (namely carbon spheres with the potential range of -1 ~ 0 V). Hence, after balancing the charges, the device assembled by two electrodes with opposite processes could work under the potential window of 0 ~ 1.4 V [46]. 2) As is shown in Fig. 6b, the CV curves of asymmetric supercapacitor show a pair of distinct peaks under the different scanning rates, which confirms the typical Faradaic pseudocapacitance characteristics contributed by NiCo-LDH-1 [47]. Furthermore, a clearer quasi-rectangular CV curve as well as an approximately linear process of charge and discharge in Fig. 6b, c for the asymmetric supercapacitor compared with that for NiCo-LDH-1 further confirms the improved capacitance responsiveness on account of the electric double-layer capacitance effect generated by the carbon spheres. Thus, the fabricated asymmetric supercapacitor exhibits a variation of the CV curve appearance relative to the single NiCo-LDH-1 and carbon spheres electrode. This results from the unification of the superiorities of two electrode materials with diverse mechanisms of charge storage. With this advantage, the voltage of the device can be improved, therefore contributing to the promotion in power and energy densities [48].

The GCD curves at different current densities are exhibited in Fig. 6c with the voltage range of 0 ~ 1.4 V. According to the calculations, the specific capacitances of asymmetric supercapacitor are 95 (132.9) and 57 F g^−1^ (79.75 C g^−1^) under the current densities of 1 and 5 A g^−1^, respectively. The maximum energy density is 25.9 W h kg^−1^ at the power density of 701.6 W kg^−1^. Meanwhile, the low-frequency curve of the EIS shown in the inset of Fig. 6d is nearly vertical, which indicates that the electrolyte ions have excellent shuttling ability between positive and negative electrodes [49]. The cycling stability of the device is also evaluated by repeated charge and discharge test at 10 A g^−1^. As shown in Fig. 6d, the capacitance retention is above 78% after 1000 cycles.

## Conclusions

A simple and effective one-step hydrothermal method is used to synthesize diverse NiCo-LDHs. The nanostructures of the NiCo-LDHs can be adjusted by optimizing the content of urea, thus improving its electrochemical performance as electrode materials. The consequences of structural characteristics show that with the decrease of urea content, the NiCo-LDH-1 nanosheets exhibit well-improved dispersity and form thin porous structure with the thickness of only 1.62 nm, which creates more active sites for redox reaction, enhances the affinity between the electrolyte and electrode material, shortens the ion diffusion path and improves electron transfer capability. The NiCo-LDH-1 displays an excellent specific capacitance of 3982.5 F g^−1^ at current density of 1 A g^−1^ and above 93.6% capacitance retention rate over 1000 cycles under the high current density of 10 A g^−1^. The excellent electrochemical performance of NiCo-LDH-1 is further attested by fabricating two-electrode asymmetric supercapacitor with carbon spheres. The specific capacitance is 95 F g^−1^ at 1 A g^−1^, and the capacitance retention is above 78% over 1000 cycles. These results indicate that the NiCo-LDHs are the next-generation energy storage material with great application prospects and provide the high possibility for the development of high-energy supercapacitors.

## Data Availability

The datasets used and/or analyzed during the current study are available from the corresponding author on reasonable request.

## Abbreviations

NiCo-LDHs: Nickel–cobalt layered double hydroxides
TMHs: Transition metal hydroxides
Ni: Nickel
Co: Cobalt
NF: Nickel foam
XRD: X-ray automatic diffractometer
XPS: X-ray photoelectron spectrometer
SEM: Scanning electron microscope
TEM: Transmission electron microscope
AFM: Atomic force microscope
EDS mapping: Energy-dispersive X-ray spectroscopy element mapping
CV: Cyclic voltammograms
GCD: Galvanostatic charge–discharge curves;
EIS: Electrochemical impedance
ASC: Asymmetric supercapacitor

## References

[1] Simon P, Gogotsi Y, Dunn B (2014). Where do batteries end and supercapacitors begin?. Mater Sci.

[2] Liu S, Li A, Han Q, Yang C, Liu X (2020) Oxygen-directed porous activation of carbon nanospheres for enhanced capacitive energy storage. J Power Sources 483:229223.

[3] Chen K, Li H, Xu Y, Liu K, Liu M (2019). Untying thioether bond structure enabled by “voltage-scissors” for stable room temperature sodium-sulfur batteries. Nanoscale.

[4] Pazhamalai P, Krishnamoorthy K, Sahoo S, Vimal K, Kim S (2019). Understanding the thermal treatment effect of two-dimensional siloxene sheets and the origin of superior electrochemical energy storage performances. ACS Appl Mater Interfaces.

[5] Qin W, Li J, Liu X, Zhou N, Wu C (2019). Formation of needle-like porous CoNi_2_S_4_-MnOOH for high performance hybrid supercapacitors with high energy density. J Colloid Interface Sci.

[6] Yang X, Liang Z, Yuan Y, Yang J, Xia H (2017) Preparation and electrochemical performance of porous carbon nanosphere. Acta Phys Sin-Ch Ed 66

[7] Yang X, Xia H, Liang Z, Li H, Yu H (2017). Monodisperse Carbon Nanospheres with Hierarchical Porous Structure as Electrode Material for Supercapacitor. Nanoscale Res Lett.

[8] Liang Z, Liu H, Zeng J, Zhou J, Li H, Xia H (2018). Facile synthesis of Nitrogen-Doped microporous carbon spheres for high performance symmetric supercapacitors. Nanoscale Res Lett.

[9] Zhang C, Cai X, Qian Y, Jiang H, Zhou L, Li B, Lai L, Shen Z, Huang W (2018). Electrochemically Synthesis of Nickel Cobalt Sulfide for High-Performance Flexible Asymmetric Supercapacitors. Advanced Science.

[10] Liang Z, Xia H, Liu H, Zhang L, Zhou J, Li H, Xie W (2019) Enhanced capacitance characteristic of microporous carbon spheres through surface modification by oxygen-containing groups. Results Phys 15:102586.

[11] Liang Z, Zhang L, Liu H, Zeng J, Zhou J, Li H, Xia H (2019). Formation of monodisperse carbon spheres with tunable size via triblock Copolymer-Assisted synthesis and their capacitor properties. Nanoscale Res Lett.

[12] Liang Z, Zhang L, Liu H, Zeng J, Zhou J, Li H, Xia H (2019). Soft-template assisted hydrothermal synthesis of size-tunable, N-doped porous carbon spheres for supercapacitor electrodes. Results in Physics.

[13] Liu S, Zhao Y, Zhang B, Xia H, Zhou J, Xie W, Li H (2018). Nano-micro carbon spheres anchored on porous carbon derived from dual-biomass as high rate performance supercapacitor electrodes. J Power Sources.

[14] Yuan R, Li H, Zhang X, Zhu H, Zhao Z (2020) Facile one-pot solvothermal synthesis of bifunctional chrysanthemum-like cobalt-manganese oxides for supercapacitor and degradation of pollutants. J Energy Storage 29:101300.

[15] Zhu X, Li X, Tao H, Li M (2021) Preparation of Co_2_Al layered double hydroxide nanosheet/Co_2_Mn bimetallic hydroxide nanoneedle nanocomposites on nickel foam for supercapacitors. J Alloys Compd 851:156868.

[16] Zhao Y, Liu S, Zhang B, Zhou J, Xie W, Li H (2018). One-step synthesis of mesoporous chlorine-doped carbonated cobalt hydroxide nanowires for high-performance supercapacitors electrode. Nanoscale Res Lett.

[17] Liu H, Liang Z, Liu S, Zhang L, Xie W, Xia H (2020) Nickel manganese hydroxides with thin-layer nanosheets and multivalences for high-performance supercapacitor. Results in Phys 16:102831.

[18] Yang Z, Ma J, Bai B, Qiu A, Shi D, Chen M (2019) Free-standing PEDOT/polyaniline conductive polymer hydrogel for flexible solid-state supercapacitors. Electrochim Acta 322:134769.

[19] Wang J, Zhang X, Wei Q, Lv H, Mai L (2015). 3D Self-Supported Nanopine Forest-Like Co_3_O_4_@CoMoO_4_ Core-Shell Architectures for High-Energy Solid State Supercapacitors. Nano Energy.

[20] Nandhini S, Muralidharan G (2021) Graphene encapsulated NiS/Ni_3_S_4_ mesoporous nanostructure: a superlative high energy supercapacitor device with excellent cycling performance. Electrochim Acta 365:137367.

[21] Wang Y, Shen C, Niu L, Li R, Guo H, Shi Y, Li C, Liu X, Gong Y (2016). Hydrothermal synthesis of CuCo_2_O_4_/CuO nanowire arrays and RGO/Fe_2_O_3_ composites for high-performance aqueous asymmetric supercapacitors. Journal of Materials Chemistry A.

[22] Cai D, Wang D, Wang C, Liu B, Wang Li, Liu Y, Li Q, Wang T (2015) Construction of desirable NiCo_2_S_4_ nanotube arrays on nickel foam substrate for pseudocapacitors with enhanced performance. Electrochim Acta 151:35–41.

[23] Cao J, Mei Q, Wu R, Wang W (2019) Flower-like nickel–cobalt layered hydroxide nanostructures for super long-life asymmetrical supercapacitors. Electrochim Acta 321:134711.

[24] Jing C, Liu X, Liu X, Jiang D, Dong B, Dong F, Wang J, Li N, Lan T, Zhang Y (2018). Crystal morphology evolution of Ni-Co layered double hydroxide nanostructure towards high-performance biotemplate asymmetric supercapacitors. Cryst Eng Comm.

[25] Cao J, Yuan S, Hao Y, Zhu Y, Chao L, Fan M, Chen H (2018). One-pot synthesis of porous nickel-manganese sulfides with tuneable compositions for high-performance energy storage. J Sol-Gel Sci Technol.

[26] Wu Z, Khalafallah D, Teng C, Wang X, Zou Q, Hong Z, Zhi M (2020) Vanadium doped hierarchical porous nickel-cobalt layered double hydroxides nanosheet arrays for high-performance supercapacitor. J Alloys Compound 838:155604.

[27] Yan T, Li R, Yang T, Li Z (2015). Nickel/cobalt layered double hydroxide hollow microspheres with hydrangea-like morphology for high-performance supercapacitors. Electrochim Acta.

[28] Ouyang Y, Xing T, Chen Y, Zheng L, Peng J, Wu C, Luo Z, Wang X (2020) Hierarchically structured spherical nickel cobalt layered double hydroxides particles grown on biomass porous carbon as an advanced electrode for high specific energy asymmetric supercapacitor. J Energy Storage 30:101454.

[29] Zha D, Fu Y, Zhang L, Zhu J, Wang X (2018). Design and fabrication of highly open nickel cobalt sulfide nanosheets on Ni foam for asymmetric supercapacitors with high energy density and long cycle-life. J Power Sources.

[30] Yan Z, Zhang H, Ling F, Ju D, Yu W (2017). Facile synthesis of nickel manganese composite oxide nanomesh for efficient oxygen evolution reaction and supercapacitors. Electrochim Acta.

[31] Xue X, Zhong J, Liu J, Hou Z, Wu X, Yu M (2020) Hydrolysis of metal-organic framework towards three-dimensional nickel cobalt-layered double hydroxide for high performance supercapacitors. J Energy Storage 31:101649

[32] Li R, Xu J, Li P, Pan Q, Ba J, Tang T, Luo W (2019). One-Step Synthesis of NiFe Layered Double Hydroxide Nanosheet Array/N-Doped Graphite Foam Electrodes for Oxygen Evolution Reactions. Chemistry Open.

[33] Shang T, Xu Y, Li P, Han J, Wu Z, Tao Y, Yang Q (2020) A bio-derived sheet-like porous carbon with thin-layer pore walls for ultrahigh-power supercapacitors. Nano Energy 70:104531.

[34] Jiang J, Sun Y, Chen Y, Zhou Q, Rong H, Hu X (2020) Design and fabrication of metal-organic frameworks nanosheet arrays constructed by interconnected nanohoneycomb-like nickel-cobalt oxide for high energy density asymmetric supercapacitors. Electrochim Acta 342:136077.

[35] Lee D, Xia Q, Mane R, Yun J, Kim K (2017). Direct successive ionic layer adsorption and reaction (SILAR) synthesis of nickel and cobalt hydroxide composites for supercapacitor applications. J Alloy Compd.

[36] Zha D, Sun H, Fu Y, Ouyang X, Wang X (2017). Acetate anion-intercalated nickel-cobalt layered double hydroxide nanosheets supported on Ni foam for high-performance supercapacitors with excellent long-term cycling stability. Electrochim Acta.

[37] Bae S, Kim J, Randriamahazaka H, Moon S, Park J, Oh I (2017). Seamlessly conductive 3D nanoarchitecture of core-shell Ni-Co nanowire network for highly efficient oxygen evolution. Adv Energy Mater.

[38] Zhang L, Cai P, Mane R, Wei Z, Liu T (2021). Synthesis of reduced graphene oxide supported nickel-cobalt-layered double hydroxide nanosheets for supercapacitors. J Colloid Interface Sci.

[39] Fang S, Li J, Xiang C, Zou Y, Xu F (2020) Anchoring sea urchin-like cobalt-nickel carbonate hydroxide on 3D carbon sponge for electrochemical energy storage. J Alloys Compd 845:156024.

[40] Tahir M, Arshad H, Xie W, Wang X, Nawaz M (2020) Synthesis of morphology controlled NiCo-LDH microflowers derived from ZIF-67 using binary additives and their excellent asymmetric supercapacitor properties. Appl Surf Scie 529:147073.

[41] Wei Z, Yuan J, Tang S, Wu D, Wu L (2019). Porous nanorods of nickel-cobalt double hydroxide prepared by electrochemical co-deposition for high-performance supercapacitors. J Colloid Interface Sci.

[42] Ge X, He Y, Plachy T, Kazantseva N, Saha P (2019). Hierarchical PANI/NiCo-LDH Core-Shell Composite Networks on Carbon Cloth for High Performance Asymmetric Supercapacitor. J Colloid Interface Sci.

[43] Song S, Zhang L, Shi H (2019). 3D nickel-cobalt double hydroxides nanosheets in situ grown on nitrogen-containing activated carbon for high-performance electrode materials. J Alloy Compd.

[44] Lv J, Yang M, Fu Y, Hideo M, Wang X (2017). The effect of urea on microstructures of tin dioxide grown on Ti plate and its supercapacitor performance. Chem Phys Lett.

[45] Azizi S, Seifi M, Randriamahazaka H, Askari M (2021) NiFe anchored to reduced graphene oxide as a low-cost and high-performance electrode material for supercapacitor applications. Phys B Condens Matter 600:412606.

[46] Yu M, Lu Y, Zheng H, Lu X (2018) New insights into the operating voltage of aqueous supercapacitors. Chem Eur J 24(15):3639–3649. 10.1002/chem.201704420 29024125

[47] Lin J, Jia H, Liang H, Chen S, Qi J (2018). In situ synthesis of vertical standing nanosized NiO encapsulated in graphene as electrodes for high-performance supercapacitors. Advanced Science.

[48] Shao Y, EI-Kady M, Sun J, Li Y (2018) Design and mechanisms of asymmetric supercapacitors. Chem Rev 118(18):9233–9280. 10.1021/acs.chemrev.8b00252 30204424

[49] Liu S, Liu K, Chen K, Fu J, Li H, Liu X (2020) Tailoring the structure of supported δ-MnO_2_ nanosheets to raise pseudocapacitance by surface-modified carbon cloth. J Power Sources 449:227507.

